# A Real-World Anti-Diabetes Medication Cost Comparison Between Premixed Insulin Analogs and Long-Acting Insulin Analogs in Chinese Patients with Type 2 Diabetes: A Retrospective Database Analysis

**DOI:** 10.1007/s13300-018-0382-8

**Published:** 2018-02-23

**Authors:** Yue Gao, Ke Wang, Yun Chen, Li Shen, Jianing Hou, Jianwei Xuan, Bao Liu

**Affiliations:** 1 Shanghai Centennial Scientific Co. Ltd, Shanghai, China; 2 Lilly Suzhou Pharmaceutical Co., Ltd, Suzhou, China; 3 Health Economic Research Institute, Sun Yat-Sen University, Guangzhou, China; 4 Department of Health Economics, School of Public Health, Fudan University, Shanghai, China

**Keywords:** China, Cost, Insulin analog, Type 2 diabetes mellitus

## Abstract

**Introduction:**

To assess and compare per-day anti-diabetic medication costs for Chinese type-2 diabetes mellitus (T2DM) insulin-naïve patients between those who initiated premixed insulin analogs (“premixed group”) and those who initiated long-acting insulin analogs (“long-acting group”).

**Methods:**

Data were obtained from an electronic medical record database between 2010.01.01 and 2015.06.30 covering medical encounter records from all general hospitals in a district from Shanghai, China. Insulin-naïve T2DM patients who were aged ≥ 18 years, treated with an oral anti-diabetic drug (OAD) only during the baseline period (3 months prior to insulin initiation), and initiated premixed or long-acting insulin analogs were included. Patients were followed until index insulin discontinuation or 12 months after initiation, whichever came first. The *t* test and generalized linear models adjusting for propensity score (PS) (including baseline demographics, number of OAD classes, comorbidities, costs, and healthcare resource utilization) were used to examine the differences between the two insulin groups.

**Results:**

A total of 570 and 185 patients were identified for the premixed and long-acting groups, with mean (SD) ages of 63.0 (12.8) and 61.1 (11.9) (*P* = 0.08) and male proportions of 47.4% and 51.4% (*P* = 0.35), respectively. During the baseline period, 19.3% of the premixed users had T2DM-related hospitalizations, while the rate was 12.4% in the long-acting group (*P* = 0.03). The mean number of T2DM-related outpatient visits was 0.98 and 1.23 for the premixed and long-acting groups, respectively (*P* = 0.07). During the follow-up period, the per-day insulin dose averaged 31.7 and 15.3 international units (IU) for the premixed and long-acting groups, respectively. Compared with the patients on premixed insulin, the mean per-day cost for patients on long-acting insulin was 37.3% higher [15.3 vs 11.2 Chinese yuan (RMB); mean difference (MD) (95% CI): 4.2 (3.2, 5.1)] for the overall anti-diabetes medication, 81.3% higher [3.3 vs 1.8 RMB; MD (95% CI): 1.5 (0.8, 2.2)] for OAD, and 28.6% higher [12.0 vs 9.3 RMB; MD (95% CI): 2.7 (2.1, 3.3)] for insulin. The results were consistent after adjusting for the PS.

**Conclusion:**

Among Chinese T2DM insulin-naïve patients, those who initiated premixed insulin had a lower per-day antidiabetic medication cost than those who initiated long-acting insulin.

**Funding:**

Lilly Suzhou Pharmaceutical Co. Ltd, China.

## Introduction

The increasing number of patients with diabetes mellitus poses a significant threat to health care systems worldwide. Based on 2015 International Diabetes Federation data, the prevalence of diabetes among people aged 20–79 years reached 8.8%. China accounts for approximately 26.5% of the 415 million diabetes mellitus patients around the world [1]. According to recent estimates, the number of patients with diabetes in China is expected to increase from 109.6 million in 2015 to 150.7 million by the year 2040 [2]. Type 2 diabetes mellitus (T2DM) is the dominant type in China, with its prevalence reaching 9.7%, accounting for more than 90% of the overall diabetes population in China [3].

Diabetes is associated with substantial medical costs in China, which exert a substantial economic impact on its national health system [5]. China’s direct medical cost of diabetes in 2004 reached 57.5 billion RMB (6.95 billion USD in 2004), accounting for 7.57% of China’s total health expenditure. In 2010, the treatment cost of diabetes in China was approximately 173.4 billion RMB (25 billion USD), accounting for approximately 13% of China’s total health expenditure; in 2015, this number surged to 51 billion USD [6].

The American Diabetes Association (ADA)/European Association for the Study of Diabetes (EASD) issued guidelines recommending long-acting insulin for patients with HbA1C > 7.0% after treatment with two oral antidiabetic drugs (OADs). Based on Chinese patient characteristics and clinical practice, Chinese treatment guidelines for T2DM in 2013 also recommended premixed insulin as initiation insulin after OAD failure [7].

In China, approximately 34% of T2DM patients are treated with insulin, and approximately two-thirds of those are on premixed insulin due to the need to control their high postprandial blood sugar levels. 27% of T2DM patients managed their diabetes with OADs and insulin [3, 6].

Insulin analogs have been widely used in China due to their advantages over traditional human insulin, such as a reduced risk of hypoglycemia, injection time flexibility, and convenience [8]. Numerous studies have compared premixed insulin analogs and long-acting insulin analogs from an efficacy and safety perspective [9]; however, in an era when the cost of clinical management has become increasingly critical in real-world clinical decision making, it is important to compare a premixed insulin analog regime with a long-acting insulin analog regime from a real-world cost perspective [6].

The purpose of this study was to assess and compare per-day antidiabetic medication costs, direct medical costs, and health resource utilization for Chinese T2DM insulin-naïve patients during the 12-month follow-up period between patients who initiated premixed insulin analogs and those who initiated long-acting insulin analogs.

## Methods

### Data Source

The Shanghai Minhang electronic medical record (EMR) database system, which is organized and supported by the Minhang Health and Family Planning Commission and covers approximately two million residents of the Minhang district of Shanghai, was the primary data source for this study. Currently, there are approximately 210,000 patients with detailed longitudinal follow-up information in this database, including demographics, diagnosis, treatments, procedures, hospitalizations, lab information, insurance status, and other related information.

A retrospective analysis was conducted using anonymized records between 1 January 2010 and 30 June 2015 from three comprehensive hospitals in the Minhang district (i.e., Fifth People’s Hospital, Wujin Hospital, and Minhang District Central Hospital).

Patients who were diagnosed with T2DM, aged ≥ 18 years, and insulin naïve (defined as not having used any type of insulin for at least three months prior to insulin initiation), had initiated either premixed or long-acting insulin at the index date, and had trackable data covering >12 months of continuous follow-up after insulin initiation were included in this study. Because the data were retrospectively extracted and only anonymized records were used in the analysis, ethical approval and written informed patient consent were not required for this study. This article does not contain any studies with human participants or animals performed by any of the authors.

Endpoints are defined and calculated as follows: per-day antidiabetic medication cost/insulin cost/OAD cost are defined as the total antidiabetic treatment cost/insulin cost/OAD cost from index insulin prescription to discontinuation divided by the number of days during the period. Discontinuation is defined as either switching to other antidiabetic medications or stopping the index insulin for > 90 days within one year after the index insulin prescription date. Index insulin persistence is defined as the time to index insulin discontinuation from the index date. OAD use is defined as any OAD used during the six months following the index date. Health care resource use is defined as inpatient and outpatient visits either for T2DM or for all-cause disease during the index insulin persistence period. Total medical cost is defined as the cost of either T2DM or all-cause disease during the index insulin persistence period.

### Statistical Analysis

The baseline characteristics of the patients were compared between the premixed and long-acting groups using the two-sample *t* test for continuous variables and the chi-square test for categorical variables. The cost and time-to-event endpoints were analyzed using a generalized linear model with a log link function and gamma distribution and the Cox proportional hazards model, respectively [10]. All unbalanced characteristics were included to calculate the propensity score (PS), and the PS was adjusted for as the covariate in the model.

A two-sided significance level of 0.05 was used. Adjustments for multiplicity were not made due to the exploratory purpose of this work. All analyses were conducted using STATA SE 12.0.

## Results

### Analyzed Population

**Fig. 1:**
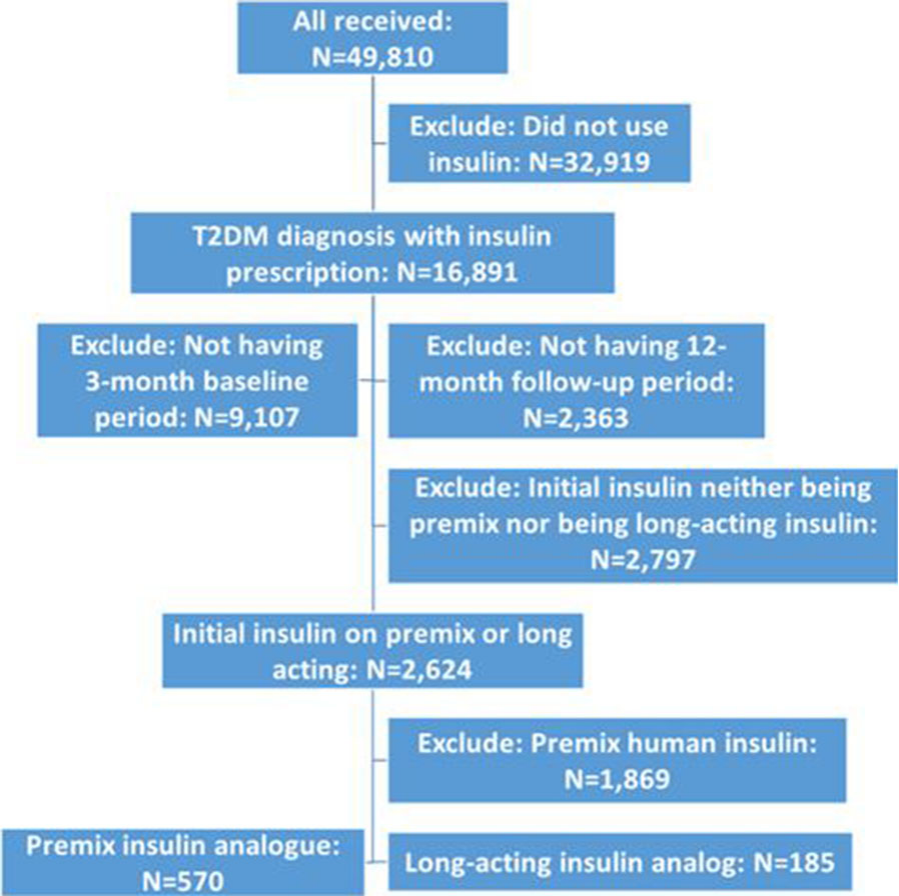
Patient flow chart

Between 1 January 2010 and 30 June 2015, a total of 16,891 patients were diagnosed with T2DM and prescribed insulin. Of these, 755 patients (570 patients initiating premixed insulin analog and 185 patients initiating long-acting insulin analog at the index date) were included in the analysis. Patient flow is detailed in Fig. 1.

### Demographics and Baseline Clinical Characteristics

There were several statistically significant differences in the baseline characteristics between the premixed and long-acting groups (Table 1). Specifically, patients in the premixed group used fewer OAD(s), had more inpatient visits for either T2DM or all causes, and spent less on medical costs for outpatient visits for either T2DM or all causes (all *P* < 0.05). Patients in the premixed group had a higher initial insulin dosage (29.6 IU) than those in the long-acting group (12.3 IU) (*P* < 0.0001) (Table 1).

**Table 1:** Demographics and baseline characteristics

	Premixed insulin (*n* = 570)	Long-acting insulin (*n* = 185)	*P* value
Age, mean (SD), years	63.0 (12.8)	61.1 (11.9)	0.0803
≥ 60, no. (%)	361.0 (63.3)	107.0 (57.8)	0.181
Female, no. (%)	300.0 (52.6)	90.0 (48.7)	0.346
Insulin dosage at index date, mean (SD), IU	29.6 (7.9)	12.3 (4.8)	< 0.0001
OAD classes, no. (%)			
0	418.0 (73.3)	97.0 (52.4)	< 0.001
1	64.0 (11.2)	28.0 (15.1)	
2	58.0 (10.2)	32.0 (17.3)	
> 2	30.0 (5.3)	28.0 (15.1)	
Inpatient visits for T2DM, no. (%)			
0	460.0 (80.7)	162.0 (87.6)	0.033
1	110.0 (19.3)	23.0 (12.4)	
Inpatient visits for all causes, no. (%)			
0	377.0 (66.1)	152.0 (82.2)	< 0.001
1	188.0 (33.0)	29.0 (15.7)	
2	5 (0.9)	3.0 (1.6)	
3	0	1.0 (0.5)	
Outpatient visits for T2DM, mean (SD), no.	1.0 (1.6)	1.2 (1.7)	0.0704
Outpatient visits for all causes, mean (SD), no.	2.7 (3.2)	2.8 (2.9)	0.6389
Total medical costs for T2DM, mean (SD), RMB	227.0 (1079.0)	327.0 (990.0)	0.2977
Total medical costs for all causes, mean (SD), RMB	276.0 (1209.0)	421.0 (1229.0)	0.2109
Total inpatient medical costs for T2DM, mean (SD), RMB	110.0 (1051.0)	103.0 (927.0)	0.935
Total inpatient medical costs for all causes, mean (SD), RMB	134.0 (1158.0)	160.0 (1146.0)	0.8114
Total outpatient medical costs for T2DM, mean (SD), RMB	121.0 (261.0)	235.0 (348.0)	< 0.0001
Total outpatient medical costs for all causes, mean (SD), RMB	159.0 (344.0)	272.0 (365.0)	0.0001

Baseline window was defined as three months before the index insulin prescription date

*OAD* oral antidiabetic drug, *T2DM* type 2 diabetes mellitus, *RMB* renminbi, the official currency of China, *SD* standard deviation of the mean

### Insulin and OAD Exposure After the Index Date

During the 12-month follow-up period, no significant difference in index insulin persistence was observed between the two groups: the treatment persistence rate was 23.5% and 31.9% and the median time to index insulin discontinuation was 113.5 and 126.7 days for the premixed group and the long-acting group, respectively (*P* = 0.07).

**Fig. 2:**
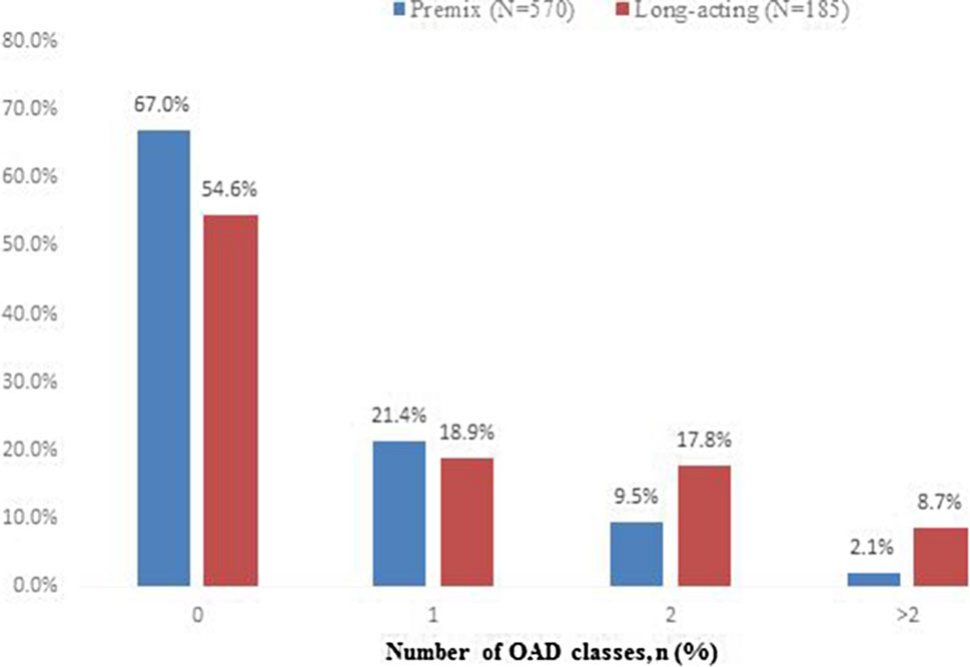
Oral antidiabetic drug exposure during the six months following insulin initiation

Patients in the premixed group injected more insulin per day (mean daily dose, 31.7 IU vs. 15.3 IU; *P* < 0.0001), and a smaller percentage of them used OADs (33.0% vs. 45.4%; *P* = 0.002) compared with the long-acting group during the six-month follow-up period (Fig. 2).

### Per-Day Cost of Antidiabetic Medications

**Fig. 3:**
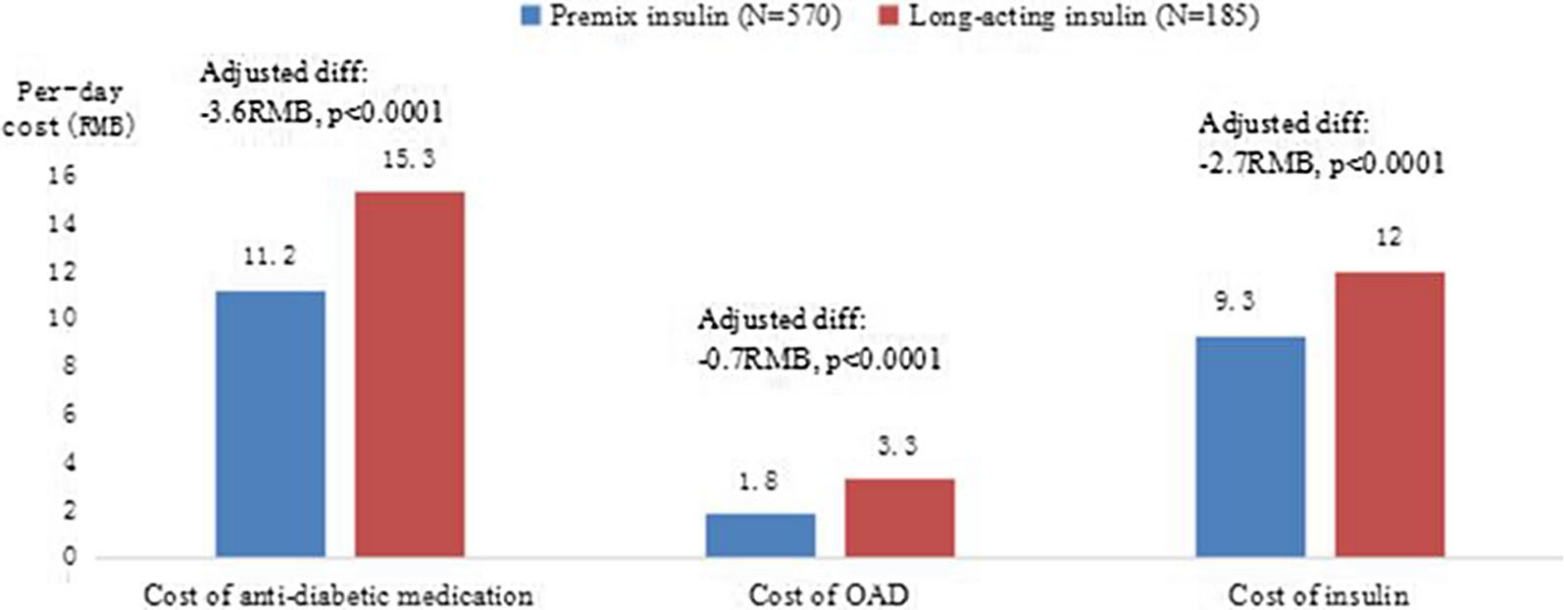
Per-day cost (in RMB)

**Fig. 4:**
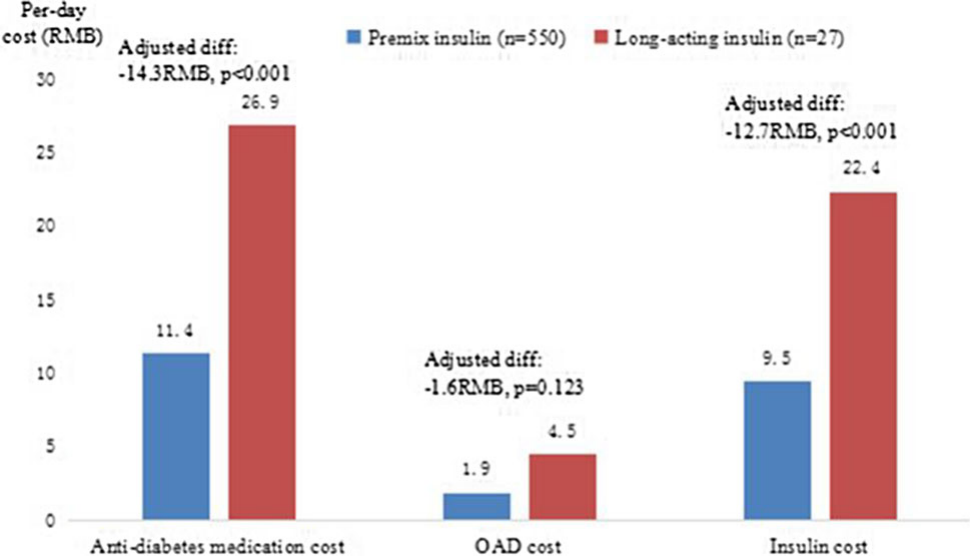
Per-day cost (in RMB) for those with a daily insulin dose of ≥ 20 IU

The mean (SD) per-day costs of antidiabetic medications, insulin, and noninsulin antidiabetic medications during the period following initiation and before discontinuation were plotted (Fig. 3). A statistically significant difference in per-day costs was observed between the two groups. The patients in the premixed group spent less on antidiabetic medications per day. The crude mean difference in per-day costs was − 4.2, − 1.5, and − 2.7 for antidiabetic medications, noninsulin antidiabetic medications, and insulin, respectively [all *P* ≤ 0.0001 (unadjusted)]. Among those requiring a higher daily insulin dose (≥ 20 IU), patients in the premixed group spent less on antidiabetic medications per day, with the cost difference between the two groups being even larger. The crude mean difference in per-day costs was − 15.5, − 2.7, and − 12.8 for antidiabetic medications, noninsulin antidiabetic medications, and insulin, respectively [all *P* ≤ 0.001 (unadjusted)] (Fig. 4).

### T2DM-Related Health Care Resource Use and Total Direct Medical Costs

During the period following initiation and before discontinuation, there was no significant difference in T2DM-related hospitalization between the two insulin groups (*P* = 0.404). The patients in the premixed group made fewer outpatient visits and spent less on total medical costs (all *P* < 0.002) compared with those in the long-acting group (Table 2).

**Table 2:** Diabetes-related resource use and total health care costs

	Premixed insulin (*n* = 570)	Long-acting insulin (*n* = 185)	Crude mean difference (95% CI)	Adjusted mean difference (95% CI)	Adjusted *P* value
Total medical costs for T2DM, mean (SE), RMB	1467.1 (67.4)	2501.2 (196.1)	− 1034.1 (− 714.7, − 1353.5)	− 946.3 (− 1374.6, − 518.0)	< 0.0001
T2DM-related outpatient visits, mean (SE), no.	6.5 (0.3)	8.3 (0.6)	− 1.8 (− 0.7, − 2.9)	− 1.5 (− 2.7, − 0.3)	0.013
Total T2DM-related outpatient medical costs, mean (SE), RMB	1467.5 (63.3)	2518.9 (193.7)	− 1051.4 (− 744.0, − 1358.8)	− 965.3 (− 1373.5, − 557.2)	< 0.0001

*CI* confidence interval, *T2DM* type 2 diabetes mellitus, *RMB*, renminbi, the official currency of China, *SE* standard error of mean, *no.* number

### Health Care Resource Use and Total Direct Medical Costs

During the period following initiation and before discontinuation, there was no significant difference in all-cause hospitalization and outpatient visits between the two insulin groups (*P* = 0.449 and *P* = 0.133). Patients in the premixed group spent less on total medical costs and less on outpatient medical costs (all *P* < 0.02) compared with those in the long-acting group (Table 3).

**Table 3:** All-cause resource use and total health care costs

	Premixed insulin (*n* = 570)	Long-acting insulin (*n* = 185)	Crude mean difference (95% CI)	Adjusted mean difference (95% CI)	Adjusted *P* value
Total medical costs, mean (SE), RMB	1894.6 (148.2)	2880.7 (228.1)	− 986.2 (− 1558.2, − 414.2)	− 877.2 (− 1649.5, − 105.0)	0.015
Outpatient visits for all cause, mean (SE), no.	9.8 (0.4)	11.3 (0.8)	− 1.5 (− 3.3, 0.2)	− 1.4 (− 3.2, 0.4)	0.133
Outpatient medical costs for all cause, mean (SE), RMB	1717.5 (70.7)	2790.2 (208.8)	− 1072.6 (− 1410.1, − 735.2)	− 1019.1 (− 1460.7, − 577.5)	< 0.001

*CI* confidence interval, *RMB* renminbi, the official currency of China, *SE* standard error of mean, *no*. number

## Discussion

Treatment cost has become an important factor in health care decision making, especially when there are many medications with similar clinical efficacies to choose from [11]. It is critical that we analyze the cost difference from a real-world perspective to provide economic evidence to support optimal clinical treatment decisions.

The advantages of premixed insulin over long-acting insulin in terms of long-term treatment cost have been demonstrated through an economic modeling methodology [5]. Our study further proves it from a real-world perspective by following two retrospective cohorts of insulin-naïve patients during the one-year period from their first use of insulin until discontinuation in order to study their respective treatment costs. We conducted this analysis with a comprehensive viewpoint from a cost perspective and considered all antidiabetic medications, which better reflects what happens in real-world diagnostics and treatment environments.

Our study results suggest that, in China, long-acting insulin users pay more (mean difference 4.17 RMB) for diabetes-related medication each day than do premixed users. The gap becomes even wider for those on higher insulin dosages. This is likely attributable to the facts that (1) the unit price is higher for long-acting insulin and (2) Chinese long-acting insulin users used more OADs as combination therapies to control postprandial blood sugar. This is consistent with our study’s finding that patients from the long-acting group used more OAD classes during both the three-month baseline period before the index insulin prescription and six months after insulin initiation compared with the premixed group.

Patients on premixed insulin injected higher doses compared with long-acting insulin users. This relates to component differences between the two kinds of insulin. Premixed delivers both long-acting (protaminated portion) and prandial (insulin lispro/insulin aspart) insulin in one injection at mealtimes [12]. Although the daily dose needed to achieve the extended effect (protaminated portion) for premixed users is comparable to that for their long-acting counterparts, its improved HbA1C reduction and better control of postprandial blood sugar contributes to the rapid-acting component of premixed insulin, which accounts for approximately 30% of the total daily dosage [9].

Our study findings also indicate that patients in the long-acting group used more OAD classes six months after insulin initiation compared with the premixed group, which may partially explain the higher per-day cost and lower insulin dosage for long-acting users; however, due to data limitations, records for patients prescribed OADs in community health care centers could not be used in our study. Therefore, true OAD usage is underestimated.

Qayyum et al. [13] conducted a systematic review of a series of published comparison studies on long-acting and premixed insulin and found that premixed analogs showed better performance in postprandial blood sugar and HbA1C reduction, whereas long-acting analogs succeeded in reducing fasting plasma glucose levels.

Glycemic control for Chinese patients with T2DM is suboptimal, because insulin therapy is initiated rather late. They tend to have early pancreatic beta-cell dysfunction and early reduction of the insulin secretion peak, leading to more severe postprandial hyperglycemia, which is made even more serious by the traditional Chinese high-carbohydrate diet. Therefore, the lifestyle of Chinese patients could be a major factor contributing to the widespread use of premixed insulin in China [4].

The calculated health care costs and resource use are underestimated. First, diabetes-related resource use (e.g., outpatient visits, inpatient visits) is defined as occurring when the patient’s first diagnosis is diabetes. However, in real clinical practice, patients with diabetes could still be prescribed an antidiabetic medication even if their first diagnosis is another disease; for example, a patient might be given hypertension as a first diagnosis and diabetes as a second diagnosis in an outpatient setting, leading to the prescription of both anti-diabetic and anti-hypertensive medications. Second, lab test expenses were not available in our database, so the true cost of health care is unknown. Third, the medication costs calculated in this study only include those associated with the study hospitals. Expenses incurred in other hospitals or pharmacies are not included, which may have resulted in an underestimation of the costs.

A broadly recognized challenge with the analysis of real-world data is the need to correct for sample selection bias [14, 15]. Generalized linear models adjusting for the propensity score, including baseline demographics, number of OAD classes, costs, and health care resource utilization, were used to improve baseline comparability between the two groups; however, missing clinical endpoints (such as HbA1C) may compromise the ability to adjust for selection bias.

Given the limitations of the current database, we do not have enough data to be able to compare the two schemes in terms of clinical effectiveness. A future study based on more data would be able to proceed further within the economic evaluation framework, leading to a more comprehensive view of the results of the comparison [15].

## Conclusion

Among Chinese insulin-naïve patients with T2DM, those who initiated premixed insulin had lower per-day antidiabetic medication costs, lower total medical costs, and lower T2DM-related medical costs (mean differences of 4.17 RMB, 877.2 RMB, and 946.3 RMB, respectively) than those who started on long-acting insulin.
